# Relevance of *PNPLA3*, *TM6SF2*, *HSD17B13*, and *GCKR* Variants to MASLD Severity in an Egyptian Population

**DOI:** 10.3390/genes15040455

**Published:** 2024-04-04

**Authors:** Nehal Elmansoury, Ahmed A. Megahed, Ahmed Kamal, Nefertiti El-Nikhely, Marina Labane, Manal Abdelmageed, Ann K. Daly, Ahmed Wahid

**Affiliations:** 1 Department of Pharmaceutical Biochemistry, Faculty of Pharmacy, Alexandria University, Alexandria 21521, Egypt; nehal.elmansoury@alexu.edu.eg; 2 Faculty of Pharmacy, Alexandria University, Alexandria 21521, Egypt; gs-ahmed.megahed@alexu.edu.eg (A.A.M.); marina.labane@gmail.com (M.L.); 3 Department of Internal Medicine and Hepatology, Faculty of Medicine, Alexandria University, Alexandria 21131, Egypt; ahmed.kamal@alexmed.edu.eg; 4 Institute of Graduate Studies and Research, Alexandria University, Alexandria 21526, Egypt; igsr.nelnikhely@alexu.edu.eg; 5 Department of Experimental and Clinical Internal Medicine, Medical Research Institute, Alexandria University, Alexandria 21561, Egypt; manal.abdelmageed@alexu.edu.eg; 6 Translational and Clinical Research Institute, Faculty of Medical Sciences, The Medical School, Newcastle University, Framlington Place, Newcastle upon Tyne NE2 4HH, UK; a.k.daly@newcastle.ac.uk

**Keywords:** non-alcoholic fatty liver disease, *PNPLA3* rs738409 C>G, *TM6SF2* rs58542926 C>T, *HSD17B13* rs9992651 G>A, *GCKR* rs1260326 T>C

## Abstract

Metabolic dysfunction-associated steatotic liver disease (MASLD), formerly known as non-alcoholic fatty liver disease (NAFLD), is a frequent clinical condition globally. Single nucleotide polymorphisms (SNPs) associated with NAFLD have been proposed in the literature and based on bioinformatic screening. The association between NAFLD and genetic variants in Egyptians is still unclear. Hence, we sought to investigate the association of some genetic variants with NAFLD in Egyptians. Egyptians have been categorized into either the MASLD group (n = 205) or the healthy control group (n = 187). The severity of hepatic steatosis and liver fibrosis was assessed by a Fibroscan device. TaqMan-based genotyping assays were employed to explore the association of selected SNPs with MASLD. *PNPLA3* rs738409 C>G variant is associated with the presence of MASLD with liver fibrosis, the severity of both hepatic steatosis and liver fibrosis, increased systolic and diastolic blood pressure and increased alanine aminotransferase (all *p* < 0.05), while the *TM6SF2* rs58542926 C>T, *HSD17B13* rs9992651 G>A, and *GCKR* rs1260326 T>C variants were not (all *p* > 0.05). The *TM6SF2* rs58542926 T allele is associated with increased fasting blood glucose and a decreased waist circumference. The *GCKR* rs1260326 C allele is associated with decreased aspartate transaminase and diastolic blood pressure (all *p* < 0.05). Only after adjusting for the risk factors (age, sex, BMI, WC, HDL, TG, diabetes mellitus, and hypertension) F2 liver fibrosis score is negatively correlated with the *HSD17B13* rs9992651 GA genotype. This study offers evidence for the association of the *PNPLA3* rs738409 C>G variant with MASLD among Egyptians and for the association of the *PNPLA3* rs738409 G allele, the *TM6SF2* rs58542926 T allele, and the *GCKR* rs1260326 C allele with some parameters of cardiometabolic criteria.

## 1. Introduction

Metabolic dysfunction-associated steatotic liver disease (MASLD), formerly known as non-alcoholic fatty liver disease (NAFLD) [1], is a widespread clinical condition that affects about a quarter of all adults globally. It is the second-most frequent cause of liver transplantation in the United States. It refers to a group of disorders that range from simple steatotic liver disease to metabolic dysfunction-associated steatohepatitis (MASH), which can lead to liver fibrosis, cirrhosis, and hepatocellular carcinoma (HCC) [2]. Although it is common in obese people, it can also occur in non-obese people [3].

Egypt is one of the top 10 countries with the highest rates of obesity in the world. According to World Health Organization (WHO) figures, almost 75% of Egyptians have a high BMI, putting them at risk for NAFLD development. More than one-third of Egyptians are affected by NAFLD, which has a direct clinical and economic burden [4]. It was shown previously in a retrospective analysis that included 2097 Egyptian patients that the most common reason for patient presentation at liver centers is NAFLD (44.9%). Unfortunately, it appears that Egyptian patients and physicians are insufficiently aware of the issue, with some patients diagnosed with NAFLD after cirrhosis has already occurred [5].

Single nucleotide polymorphisms (SNPs) have been proposed to identify the genetic association with different diseases and their complications. SNPs associated with NAFLD have been proposed in the literature and based on bioinformatic screening. Strong evidence for the heredity of NAFLD comes from epidemiology, family aggregation, and data from twin studies. Heritability estimates range from 20% to 70% based on ethnicity, study design, environmental factors, and the methodologies used to characterize NAFLD [6]. The first gene variant found to have a consistent genetic association with NAFLD was the patatin-like phospholipase domain containing 3 (*PNPLA3* rs738409 C>G) [7], and this association has been replicated extensively [8,9,10,11,12,13,14,15,16,17,18,19]. PNPLA3-induced steatohepatitis patients are known to be associated with developing liver fibrosis, cirrhosis, and, ultimately, HCC. In addition, other studies have shown that transmembrane 6 superfamily 2 (*TM6SF2* rs58542926 C>T) and hydroxysteroid 17-beta dehydrogenase 13 (*HSD17B13* rs9992651 G>A) are also associated with NAFLD development [18,20,21,22]. Another study revealed that the *TM6SF2* rs58542926 C>T variant shows no significant correlation with NAFLD [23]. Some studies also show a role for the glucokinase regulatory protein in NAFLD (*GCKR* rs1260326 T>C) [18,24]. The genetic association with NAFLD may be modified by the change in the population [7,14,25,26]. The association between NAFLD and genetic variants in Egyptian subjects is still unclear. Hence, we sought to investigate the association of these genetic variants with NAFLD in Egyptian patients.

## 2. Patients and Methods

### 2.1. Bioinformatic Analysis

A protein–protein interaction network was generated using the STRING web-based tool to verify the interaction between the selected genes [27].

Prediction of the impact of the selected genetic variants on protein function was performed using the SIFT 6.2.1 predictive tool [28,29].

Homology modeling was performed using the SWISS-MODELworkspace to generate a 3D model of PNPLA3. Two models were generated using the template PDB ID: Q9NST1.1.A, one with position 148 as Isoleucine and another with the variant p.Ile148Met. Pymol (The PyMOL Molecular Graphics System, Version 3.0) was used to visualize the models and measure the polar distances at position 148 [30].

To evaluate the relationship between selected sequences, a phylogenetic analysis was performed using the UPGMA method in MEGA X (Version 10.1.8) software. The tree is drawn to scale, and the evolutionary distances were computed using the Poisson correction method and are in units of the number of amino acid substitutions per site [31].

### 2.2. Patients

Egyptian patients who participated in this study were recruited from Alexandria University hospitals between 1 January 2021 and 1 December 2022. A total of 392 participants signed a written consent document. These individuals have been categorized into either the MASLD group (n = 205) or the healthy control group (n = 187). A subgroup of 131 individuals with liver fibrosis in MASLD was compared with 187 control individuals; age, sex, waist circumference (WC), body mass index (BMI), systolic blood pressure (SBP), and diastolic blood pressure (DBP) were included in the information.

Biochemical analysis: plasma aspartate aminotransferase (AST), alanine aminotransferase (ALT), gamma-glutamyl transferase (GGT), fasting blood sugar (FBS), post-prandial blood sugar (PPBS), total cholesterol (TC), serum low-density lipoprotein cholesterol (LDL-C), serum high-density lipoprotein cholesterol (HDL-C), and triglycerides (TG) were measured for all participants [32].

Cases were defined as having steatotic liver disease (SLD) based on the evidence of steatotic liver using a Fibroscan device. Patients with controlled attenuation parameter (CAP) scores equal to or greater than 238 dB/m (decibels per meter) were included. The presence of at least one out of five cardiometabolic adult criteria defines patients who are currently diagnosed with MASLD. Other causes of chronic liver diseases, including alcohol intake, drug-induced fatty liver, chronic viral hepatitis B and C, autoimmune hepatitis, hemochromatosis, α1-antitrypsin deficiency, Wilson’s disease, and Celiac disease, were excluded from our study.

Cardiometabolic criteria of the study population: (1) BMI ≥ 25 kg/m^2^ or WC > 94 cm for males (M) and 80 cm for females (F), (2) FBG ≥ 100 mg/dL or PPBS ≥ 140 mg/dL or treatment for type 2 diabetes, (3) BP ≥ 130/85 mmHg or specific antihypertensive drug treatment, (4) TG ≥ 150 mg/dL or lipid-lowering treatment, (5) HDL-C ≤ 40 mg/dL (M) and ≤50 mg/dL (F) or lipid-lowering treatment (1).

Controls were defined as having no SLD based on no evidence of steatotic liver using a Fibroscan device. Healthy subjects with CAP scores less than 238 dB/m, normal liver enzymes and functions, and a normal liver on ultrasonography were included. The same exclusion criteria were applied to the control group.

#### 2.2.1. Evaluation of the Stages of Hepatic Steatosis and Liver Fibrosis

According to the manufacturer’s guidelines, we used a Fibroscan device (FibroScan^®^ Mini+ 430 Model by Echosens manufacturer, Paris, France), which can measure a volume of liver tissue 100 times the size of a liver biopsy, which minimizes sampling error [33]. A controlled attenuation parameter (CAP) scores less than 238 dB/m (decibels per meter)-denotes a healthy liver with no steatosis, 238 to 260 dB/m, 260 to 290 dB/m, and 290 dB/m or more indicate, S1, S2, and S3, respectively. A liver with no or minimal scarring has a fibrosis score of F0 to F1 (2 to 7 kPa). The F2 fibrosis score (7.5 to 10 kPa) indicates a considerable amount of scarring that has extended outside the liver. The F3 fibrosis score (10 to 14 kPa) denotes extensive scarring that has spread and interferes with normal blood flow; the F4 fibrosis score (14 kPa or more) indicates late-stage scarring, often known as cirrhosis, in which the damage and the scarring are irreversible [34].

#### 2.2.2. Genotyping

Genomic DNA extraction from blood samples was performed using a QIAamp (Qiagen, Hilden, Germany) DNA Blood Mini Kit, following the manufacturer’s instructions. To evaluate the extracted DNA qualitatively and quantitatively, we used a Thermo Scientific Nanodrop 2000 spectrophotometer, Wilmington, NC, USA. Each DNA sample underwent genotyping analysis for the variants *PNPLA3* rs738409 C>G, *TM6SF2* rs58542926 C>T, *HSD17B13* rs9992651 G>A, and *GCKR* rs1260326 T>C. Using TaqMan SNP Genotyping Assays (ThermoFisher, Waltham, MA, USA), the experiment was conducted on a QuantStudio Real-Time PCR System (Applied Biosystems, ThermoFisher, Waltham, MA, USA). TaqMan Genotype Software (Applied Biosystems, QuantStudio^TM^ Design & Analysis Software v1.4.1) was used to analyze these data for genotype, and allelic discrimination plots were created to show the genotypes of all samples.

### 2.3. Statistical Analysis

The statistical tests utilized were the Chi-square test to contrast various groupings using categorical variables, Monte Carlo or Fisher’s exact correction to correct Chi-square when more than 20% of the cells have an expected count that is less than 5, the Mann–Whitney test to compare two groups under study for quantitative variables with aberrant distributions, and the Kolmogorov–Smirnov test to examine the normality of the distribution. Regression analysis was used, and adjusted model regression analysis was used to correct age, sex, BMI, WC, HDL, TG, diabetes mellitus, and hypertension.

Confidence intervals (CI) set to 95% with odds ratios (OR) were used to express multivariable studies. These data were analyzed using the IBM SPSS software package, version 20.0. (Armonk, NY, USA: IBM Corp.). Qualitative data were described by numbers and percentages. Quantitative data were characterized by the range (minimum and maximum), mean, standard deviation, median, and interquartile range (IQR). At the 5% level, the statistical significance of the results was determined.

A power calculation of sample size to evaluate the effect of genetic variations in MASLD was performed using Gpower 3.1.9.4 software. These power calculations are summarised in Table S9. False Discovery Rate (FDR) correction for multiple comparisons was performed using the Benjamini and Hochberg method. The control group for each SNP was explored to find its equilibrium with the Hardy–Weinberg Equilibrium (HWE) using HW_TEST software v.1.1 [35]. These HWE calculations are in Table S1.

## 3. Results

### 3.1. Bioinformatic Analyses

Using the Genome-wide association studies (GWAS) catalog, four SNPs associated with MASLD were selected, namely *PNPLA3* rs738409 C>G, *TM6SF2* rs58542926 C>T, *HSD17B13* rs9992651 G>A, and *GCKR* rs1260326 T>C, and ranked based on their *p*-value (Figure S1A). The protein TM6SF2 is co-expressed with GCKR and HSD17B13, whereas PNPLA3 is only co-expressed with GCKR (Figure S1B). From the four selected SNPs, only rs738409 C>G in *PNPLA3* was predicted to be deleterious as it has a substitution score < 0.05 and causes the substitution of isoleucine to methionine. The SNPs rs58542926 C>T and rs1260326 T>C in *TM6SF2* and *GCKR*, respectively, were predicted to be tolerated, though they did not result in a silent mutation. It is worth mentioning that rs9992651 G>A in *HSD17B13* is an intron variant with no known effects on the protein (Figure S1C).

The *PNPLA3* rs738409 C>G G allele results in a non-synonymous substitution (Figure S2). Multiple sequence alignments of PNPLA3 among different species (such as mice and rats as well as in common domestic animals) revealed that position 148 (isoleucine) is highly conserved (Figure S3A). The phylogenetic analysis revealed an average similarity of 76% (Figure S3B) upon comparing the human PNPLA3 protein sequence with the sequence of common orthologues.Distance calculations showed that having a methionine at position 148 increased the polar distances (Figure S4).

### 3.2. Baseline Characteristics of the Study Population

All cases were diagnosed with MASLD. A total of 392 participants were categorized into either the MASLD group (n = 205) or the healthy control group (n = 187). The clinical, demographic, and biochemical characteristics of the participants are listed in Table 1. Compared with those without MASLD, participants with MASLD were found to be older, diabetic or prediabetic, hypertensive, more likely to be male, and had higher hepatic steatosis degree, liver fibrosis degree, body mass index (BMI), waist circumference (WC), systolic blood pressure (SBP), diastolic blood pressure (DBP), alanine aminotransferase (ALT), aspartate transaminase (AST), gamma-glutamyl transpeptidase (GGT), fasting blood glucose (FBG), post-prandial blood sugar (PPBS), total cholesterol (TC), triacylglyceride (TG), and low-density lipoprotein cholesterol (LDL-C) levels, yet lower levels of high-density lipoprotein cholesterol (HDL-C) (all *p* < 0.05).

### 3.3. Genotype Data

#### 3.3.1. The Proportions of Participants with Genotypes and Alleles of *PNPLA3* rs738409 C>G, *TM6SF2* rs58542926 C>T, *HSD17B13* rs9992651 G>A, and *GCKR* rs1260326 T>C in MASLD Cases and Controls

**Figure 1:**
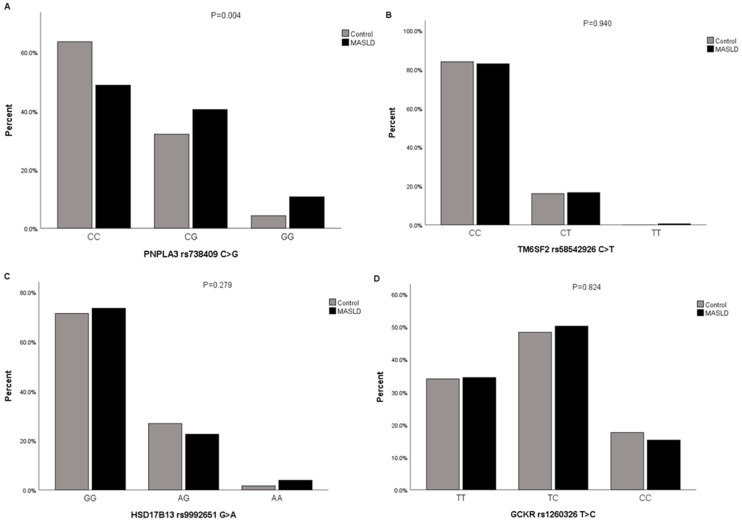
The proportions of participants with genotypes of *PNPLA3* rs738409 C>G, *TM6SF2* rs58542926 C>T, *HSD17B13* rs9992651 G>A, and *GCKR* rs1260326 T>C in subjects with MASLD and controls. (**A**) The proportions of participants with genotypes of *PNPLA3* rs738409 C>G in subjects with MASLD and controls; (**B**) The proportions of participants with genotypes of *TM6SF2* rs58542926 C>T in subjects with MASLD and controls; (**C**) The proportions of participants with genotypes of *HSD17B13* rs9992651 G>A in subjects with MASLD and controls; (**D**) The proportions of participants with genotypes of *GCKR* rs1260326 T>C in subjects with MASLD and controls, *p*-value estimated by Chi-Square or Fisher’s Exact tests for categorical datasets.

The proportions of participants with and without MASLD in different genotypes of *PNPLA3* rs738409 C>G were assessed (*p* = 0.004, FDR corrected *p*-value = 0.016). CC genotype carriers in MASLD cases had a lower proportion than those in the controls (48.8% vs. 63.6%), CG genotype carriers in MASLD cases had a higher proportion than those in the controls (40.5% vs. 32.1%), GG genotype carriers in MASLD cases had a higher proportion than those in the controls (10.7% vs. 4.3%). However, no statistically significant differences in the *TM6SF2* rs58542926 C>T, *HSD17B13* rs9992651 G>A, and *GCKR* rs1260326 T>C genotypes were observed among the groups (Figure 1A–D) (Table S1).

The proportions of participants with and without MASLD in different alleles of *PNPLA3* rs738409 C>G were also assessed (*p* = 0.001). C allele carriers in MASLD cases had a lower proportion than those in the controls (69% vs. 79.7%), and G allele carriers in MASLD cases had a higher proportion than those in the controls (31% vs. 20.3%); however, no statistically significant differences in the *TM6SF2* rs58542926 C>T, *HSD17B13* rs9992651 G>A, and *GCKR* rs1260326 T>C alleles were observed among the groups (Table S1).

#### 3.3.2. Association of Genotypes of *PNPLA3* rs738409 C>G, *TM6SF2* rs58542926 C>T, *HSD17B13* rs9992651 G>A, and *GCKR* rs1260326 T>C with MASLD by Logistic Regression Analysis

A single logistic regression analysis (Table 2) for the association of *PNPLA3* rs738409 C>G with MASLD showed statistical significance with an increased odds ratio. MASLD was positively correlated with the GG genotype (OR = 3.272, 95%CI 1.396–7.670, *p* = 0.006) and the CG genotype (OR = 1.646, 95%CI 1.076–2.519, *p* = 0.022). Single logistic regression analyses for *TM6SF2* rs58542926 C>T, *HSD17B13* rs9992651 G>A, and *GCKR* rs1260326 T>C did not show statistical significance.

After adjusting for the risk factors (age, sex, BMI, WC, HDL, TG, diabetes mellitus, and hypertension) (Table 2), MASLD was positively correlated only with the GG genotype (OR = 9.166, 95%CI 1.123–74.832, *p* = 0.039). Analysis after correction for the risk factors for *TM6SF2* rs58542926 C>T, *GCKR* rs1260326 T>C and *HSD17B13* rs9992651 G>A did not show statistical significance.

#### 3.3.3. The Proportions of Participants with Genotypes of *PNPLA3* rs738409 C>G, *TM6SF2* rs58542926 C>T, *HSD17B13* rs9992651 G>A, and *GCKR* rs1260326 T>C in MASLD Cases with Liver Fibrosis, MASLD Cases without Liver Fibrosis, and Controls

**Figure 2:**
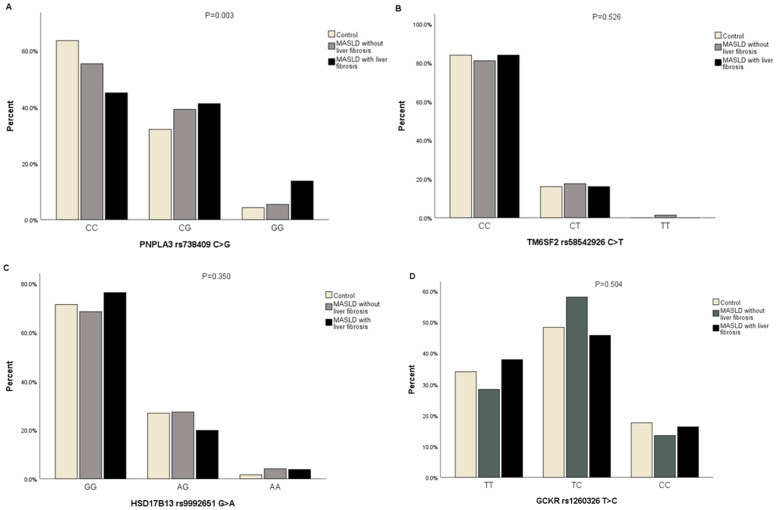
The proportions of participants with genotypes of *PNPLA3* rs738409 C>G, *TM6SF2* rs58542926 C>T, *HSD17B13* rs9992651 G>A, and *GCKR* rs1260326 T>C in cases with and without liver fibrosis in MASLD and controls. (**A**) The proportions of participants with genotypes of *PNPLA3* rs738409 C>G in cases with and without liver fibrosis in MASLD and controls; (**B**) The proportions of participants with genotypes of *TM6SF2* rs58542926 C>T in cases with and without liver fibrosis in MASLD and controls.; (**C**) The proportions of participants with genotypes of *HSD17B13* rs9992651 G>A in cases with and without liver fibrosis in MASLD and controls; (**D**) The proportions of participants with genotypes of *GCKR* rs1260326 T>C in cases with and without liver fibrosis in MASLD and controls, *p*-value estimated by Chi-Square or Fisher’s Exact tests for categorical datasets.

The proportions of cases with and without liver fibrosis in MASLD and controls in different genotypes of *PNPLA3* rs738409 C>G were assessed (*p* = 0.003, FDR corrected *p*-value = 0.012). CC genotype carriers with liver fibrosis in MASLD cases had a lower proportion than those in cases without liver fibrosis and controls (45% vs. 55.4% vs. 63.6%); CG genotype carriers with liver fibrosis in MASLD cases had a higher proportion than those in cases without liver fibrosis and controls (41.2% vs. 39.2 vs. 32.1%). Moreover, GG genotype carriers with liver fibrosis in MASLD cases had a higher proportion than those in cases without liver fibrosis and controls (13.7% vs. 5.4 vs. 4.3%). However, no statistically significant differences in *TM6SF2* rs58542926 C>T, *HSD17B13* rs9992651 G>A, and *GCKR* rs1260326 T>C genotypes were observed among the groups (Figure 2A–D) (Table S2).

The proportions of cases with and without liver fibrosis in MASLD and controls in different alleles of *PNPLA3* rs738409 C>G were also assessed (*p* < 0.001). C allele carriers with liver fibrosis in MASLD cases had a lower proportion than those in cases without liver fibrosis and controls (65.6% vs. 75% vs. 79.7%), G allele carriers with liver fibrosis in MASLD cases had a higher proportion than those in cases without liver fibrosis and controls (34.4% vs. 25% vs. 20.3%). However, no statistically significant differences in the *TM6SF2* rs58542926 C>T, *HSD17B13* rs9992651 G>A, and *GCKR* rs1260326 T>C alleles were observed among the groups (Table S2).

#### 3.3.4. Association of Genotypes of *PNPLA3* rs738409 C>G, *TM6SF2* rs58542926 C>T, *HSD17B13* rs9992651 G>A, and *GCKR* rs1260326 T>C with MASLD with Liver Fibrosis and MASLD without Liver Fibrosis

Logistic regression analysis (Table 3) for the association of *PNPLA3* rs738409 C>G with MASLD with liver fibrosis and MASLD without liver fibrosis was assessed. MASLD with liver fibrosis was positively correlated with the GG genotype (OR = 4.538, 95%CI 1.865–11.044, *p* = 0.001) and the CG genotype (OR = 1.815, 95%CI 1.121–2.940, *p* = 0.015). MASLD without liver fibrosis did not show statistical significance with the GG and CG genotypes. Logistic regression analysis for *TM6SF2* rs58542926 C>T, *HSD17B13* rs9992651 G>A, and *GCKR* rs1260326 T>C did not show statistical significance.

After adjusting for the risk factors (age, sex, BMI, WC, HDL, TG, diabetes mellitus, and hypertension) (Table S3), the association of *PNPLA3* rs738409 C>G, MASLD without liver fibrosis was positively correlated with the GG genotype (OR = 9.085, 95%CI 1.073–76.885, *p* = 0.043), while the CG genotype did not show statistical significance. MASLD with liver fibrosis did not show statistical significance with the GG and CG genotypes. *TM6SF2* rs58542926 C>T, *HSD17B13* rs9992651 G>A and *GCKR* rs1260326 T>C did not show statistical significance.

#### 3.3.5. The Proportions of Participants with Genotypes of *PNPLA3* rs738409 C>G, *TM6SF2* rs58542926 C>T, *HSD17B13* rs9992651 G>A, and *GCKR* rs1260326 T>C in Subjects with Different Severities of Liver Fibrosis

**Figure 3:**
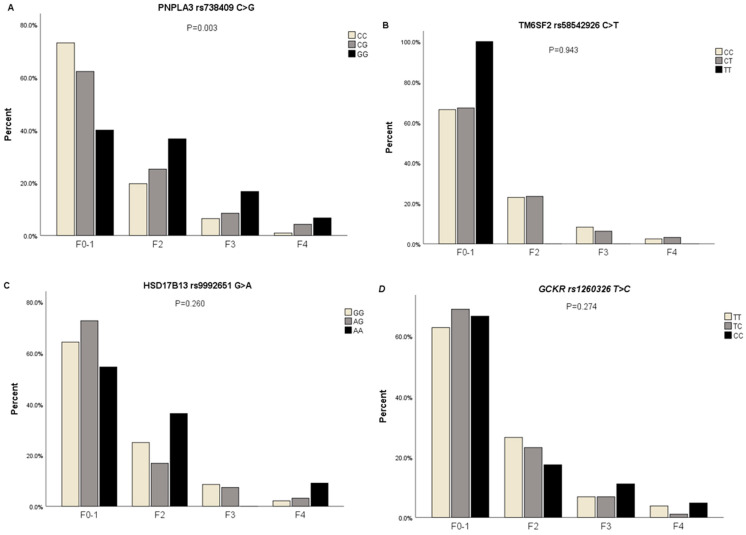
The proportions of participants with genotypes of *PNPLA3* rs738409 C>G, *TM6SF2* rs58542926 C>T, *HSD17B13* rs9992651 G>A, and *GCKR* rs1260326 T>C in subjects with different severities of liver fibrosis. (**A**) The proportions of participants with genotypes of *PNPLA3* rs738409 C>G in subjects with different severities of liver fibrosis; (**B**) The proportions of participants with genotypes of *TM6SF2* rs58542926 C>T in subjects with different severities of liver fibrosis; (**C**) The proportions of participants with genotypes of *HSD17B13* rs9992651 G>A in subjects with different severities of liver fibrosis; (**D**) The proportions of participants with genotypes of *GCKR* rs1260326 T>C in subjects with different severities of liver fibrosis, *p*-value estimated by Chi-Square or Fisher’s Exact tests for categorical datasets.

Participants were divided into F0–1, F2, F3, and F4 according to the fibrosis score using the Fibroscan device. The proportions of participants with different severities of liver fibrosis in different genotypes of *PNPLA3* rs738409 C>G were assessed (*p* = 0.003, FDR corrected *p*-value = 0.012). CC genotype carriers in subjects with F0–1 liver fibrosis had a higher proportion than CG and GG genotype carriers (73% vs. 62.2% vs. 40%), CC genotype carriers in subjects with F2 liver fibrosis had a lower proportion than CG and GG genotype carriers (19.6% vs. 25.2% vs. 36.7%), CC genotype carriers in subjects with F3 liver fibrosis had a lower proportion than CG and GG genotype carriers (6.4% vs. 8.4% vs. 16.7%), and CC genotype carriers in subjects with F4 liver fibrosis had a lower proportion than CG and GG genotype carriers (1% vs. 4.2% vs. 6.7%). However, no statistically significant differences in *TM6SF2* rs58542926 C>T, *HSD17B13* rs9992651 G>A, and *GCKR* rs1260326 T>C genotypes were observed among the groups (Figure 3A–D) (Table S4).

#### 3.3.6. Association of Genotypes of *PNPLA3* rs738409 C>G, *TM6SF2* rs58542926 C>T, *HSD17B13* rs9992651 G>A, and *GCKR* rs1260326 T>C with Different Severities of Liver Fibrosis in MASLD by Logistic Regression Analysis

Logistic regression analysis (Table 4) for the association of *PNPLA3* rs738409 C>G with different severities of liver fibrosis in MASLD was assessed. F2 was positively correlated with the GG genotype (OR = 3.411, 95%CI 1.408–8.262, *p* = 0.007), F3 was positively correlated with the GG genotype (OR = 4.762, 95%CI 1.467–15.460, *p* = 0.009), F4 was positively correlated with the GG genotype (OR = 13.333, 95%CI 1.724–103.145, *p* = 0.013), F2-F3 did not show statistical significance with the CG genotype, and F4 was positively correlated with the CG genotype (OR = 5.393, 95%CI 1.066–27.284, *p* = 0.042). Logistic regression analysis for *TM6SF2* rs58542926 C>T, *HSD17B13* rs9992651 G>A, and *GCKR* rs1260326 T>C did not show statistical significance.

After adjusting for the risk factors (age, sex, BMI, WC, HDL, TG, diabetes mellitus, and hypertension) (Table S5), the association of *PNPLA3* rs738409 C>G, F2 was positively correlated with the GG genotype (OR = 5.482, 95%CI 1.572–19.112, *p* = 0.008), F3 was positively correlated with the GG genotype (OR = 9.641, 95%CI 1.493–62.233, *p* = 0.017), F4 was positively correlated with the GG genotype (OR = 107.296, 95%CI 1.411–8157.152, *p* = 0.034), F2-F3-F4 did not show statistical significance with the CG genotype. For the association of *HSD17B13* rs9992651 G>A, F2 was negatively correlated with the GA genotype (OR = 0.341, 95%CI 0.148–0.787, *p* = 0.012). *TM6SF2* rs58542926 C>T and *GCKR* rs1260326 T>C did not show statistical significance.

#### 3.3.7. The Proportions of Participants with Genotypes of *PNPLA3* rs738409 C>G, *TM6SF2* rs58542926 C>T, *HSD17B13* rs9992651 G>A, and *GCKR* rs1260326 T>C in Subjects with Different Severities of Hepatic Steatosis

**Figure 4:**
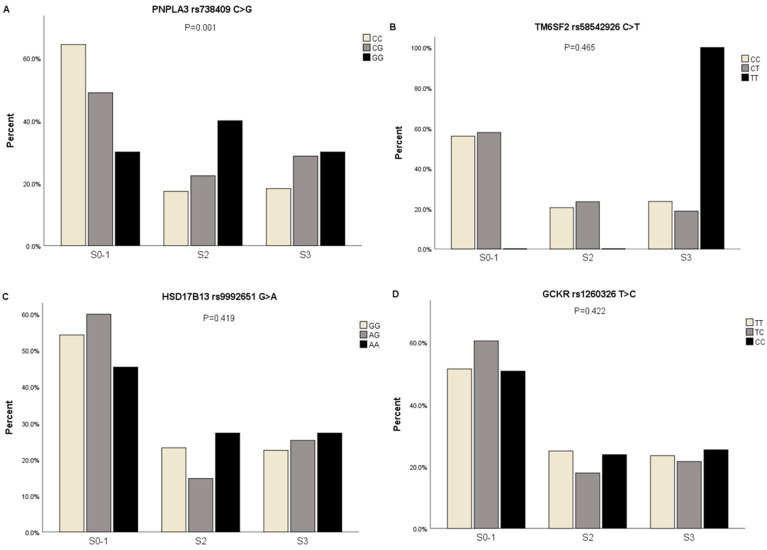
The proportions of participants with genotypes of *PNPLA3* rs738409 C>G, *TM6SF2* rs58542926 C>T, *HSD17B13* rs9992651 G>A, and *GCKR* rs1260326 T>C in subjects with different severities of hepatic steatosis. (**A**) The proportions of participants with genotypes of *PNPLA3* rs738409 C>G in subjects with different severities of hepatic steatosis; (**B**) The proportions of participants with genotypes of *TM6SF2* rs58542926 C>T in subjects with different severities of hepatic steatosis; (**C**) The proportions of participants with genotypes of *HSD17B13* rs9992651 G>A in subjects with different severities of hepatic steatosis; (**D**) The proportions of participants with genotypes of *GCKR* rs1260326 T>C in subjects with different severities of hepatic steatosis, *p*-value estimated by Chi-Square or Fisher’s Exact tests for categorical datasets.

Participants were divided into S0-1, S2, and S3 according to the CAP score using the Fibroscan device. The proportions of cases with different severities of hepatic steatosis in different genotypes of *PNPLA3* rs738409 C>G were assessed (*p* = 0.001, FDR corrected *p*-value = 0.004). CC genotype carriers in subjects with S0-1 hepatic steatosis had a higher proportion than CG and GG genotype carriers (64.4% vs. 49% vs. 30%), CC genotype carriers in subjects with S2 hepatic steatosis had a lower proportion than CG and GG genotype carriers (17.4% vs. 22.4% vs. 40%), and CC genotype carriers in subjects with S3 hepatic steatosis had a lower proportion than CG and GG genotype carriers (18.3% vs. 28.7% vs. 30%). However, no statistically significant differences in *TM6SF2* rs58542926 C>T, *HSD17B13* rs9992651 G>A, and *GCKR* rs1260326 T>C genotypes were observed among the groups (Table S6) (Figure 4A–D).

#### 3.3.8. Association of Genotypes of *PNPLA3* rs738409 C>G, *TM6SF2* rs58542926 C>T, *HSD17B13* rs9992651 G>A, and *GCKR* rs1260326 T>C with Different Severities of Hepatic Steatosis in MASLD by Logistic Regression Analysis

Logistic regression analysis (Table S7) for the association of *PNPLA3* rs738409 C>G with different severities of hepatic steatosis in MASLD was assessed, S2 was positively correlated with the GG genotype (OR = 4.947, 95%CI 1.941–12.609, *p* = 0.001), S3 was positively correlated with the GG genotype (OR = 3.525, 95%CI 1.312–9.471, *p* = 0.012), S2 did not show statistical significance with the CG genotype, and S3 was positively correlated with the CG genotype (OR = 2.065, 95%CI 1.226–3.478, *p* = 0.006). Logistic regression analysis for *TM6SF2* rs58542926 C>T, *HSD17B13* rs9992651 G>A, and *GCKR* rs1260326 T>C did not show statistical significance.

#### 3.3.9. Clinical Characteristics of *PNPLA3* rs738409 G, *TM6SF2* rs58542926 T, *HSD17B13* rs9992651 A, and *GCKR* rs1260326 C Carriers and Non-Carriers in the Study Population

The *PNPLA3* rs738409 G allele is associated with increased systolic blood pressure (*p* = 0.029, FDR corrected *p*-value = 0.145), diastolic blood pressure values (*p* = 0.015, FDR corrected *p*-value = 0.113), and alanine aminotransferase levels (*p* = 0.002, FDR corrected *p*-value = 0.030). The *TM6SF2* rs58542926 T allele is associated with increased fasting blood glucose levels (*p* = 0.017, FDR corrected *p*-value = 0.128). Although the waist circumference value is significantly higher in MASLD patients compared with the controls, the *TM6SF2* rs58542926 T allele is associated with a decreased value of waist circumference (*p* = 0.013, FDR corrected *p*-value = 0.128). Although the aspartate transaminase levels and diastolic blood pressure values are significantly higher in MASLD patients compared with the controls, the *GCKR* rs1260326 C allele is associated with decreased aspartate transaminase levels (*p* = 0.014, FDR corrected *p*-value = 0.210) and diastolic blood pressure values (*p* = 0.043, FDR corrected *p*-value = 0.236). Other clinical characteristics did not show statistically significant differences between carriers and non-carriers in the study population (Table S8A,B).

## 4. Discussion

MASLD, formerly known as NAFLD, is a silent disease affecting about a quarter of all adults globally. GWAS validates *PNPLA3* rs738409 C>G, with significant contributions from *TM6SF2* rs58542926 C>T, *HSD17B13* rs9992651 G>A, and *GCKR* rs1260326 T>C, as a risk factor for the entire histological spectrum of NAFLD at genome-wide significance levels [18,36]. *PNPLA3* rs738409 C>G had the best correlation to NAFLD. Despite not being on the same chromosome, these genes are seen to be linked, as seen in the predicted protein–protein interaction network using a string database [27]. The protein TM6SF2 is co-expressed with GCKR and HSD17B13, whereas PNPLA3 is only co-expressed with GCKR. However, all four proteins were related to NAFLD. Hence, SNPs in any of these genes might affect the expression levels of these genes. Using the SIFT web-based tool, of the four selected SNPs, only rs738409 C>G in *PNPLA3* was predicted to be deleterious as it has a substitution score < 0.05 and causes the substitution of isoleucine to methionine. The SNPs rs58542926 C>T and rs1260326 T>C in *TM6SF2* and *GCKR*, respectively, were predicted to be tolerated, though they did not result in a silent mutation. It is worth mentioning that rs9992651 G>A in *HSD17B13* is an intron variant with no known effects on the protein. Previously, there was a study showing that adiposity significantly increases the effect of the three variants (PNPLA3-I148M, TM6SF2-E167K, and GCKR-P446L) associated with nonalcoholic fatty liver disease (NAFLD).

The genetic association with NAFLD may be modified by the change in the population [7,14,25,26]. As yet, studies on the association of *PNPLA3* rs738409 C>G, *TM6SF2* rs58542926 C>T, *HSD17B13* rs9992651 G>A, and *GCKR* rs1260326 T>C with NAFLD in the Egyptian population are still limited. To the best of our knowledge, our current study is the first to investigate the association of *PNPLA3* rs738409 C>G, *TM6SF2* rs58542926 C>T, *HSD17B13* rs9992651 G>A, and *GCKR* rs1260326 T>C SNPs with clinical characteristics, the presence and severity of hepatic steatosis, and liver fibrosis in MASLD in the Egyptian population.

PNPLA3 is a triacylglycerol lipase that mediates the hydrolysis of triglycerides in adipocytes. Several studies have proven that the PNPLA3-I148M shows a modest decrease in lipolytic activity [37]. The *PNPLA3* rs738409 C>G genetic variation is located in Exon 3 of the *PNPLA3* gene. In other populations, all four nucleotides are represented. However, only the G allele results in a non-synonymous substitution. Though the genetic variant p.Ile148Met is not located within the active site, multiple sequence alignments of PNPLA3 among different species revealed that position 148 is highly conserved. In common model organisms such as mice and rats, as well as in common domestic animals, amino acid 148 was isoleucine, suggesting an important function in this position. The phylogenetic analysis revealed a similarity of 76%. After comparing the 3D structures of the PNPLA3 protein with those of Ile148 and Met148, distance calculations showed that having a methionine at position 148 increased the polar distances, thus hampering possible interactions that might affect protein conformation and ligand binding.

The first gene variant found to have a consistent genetic association with NAFLD was *PNPLA3* rs738409 C>G [7], and this association has been replicated extensively [8,9,10,11,12,13,14,15,16,17,18,19]. PNPLA3-induced steatohepatitis patients are known to be associated with developing liver fibrosis, cirrhosis, and, ultimately, HCC. The results of the current study agree with a meta-analysis of 16 studies [11] that revealed an association between the *PNPLA3* rs738409 C>G polymorphism and NAFLD, as well as the development of fibrosis in different populations around the world, not including the Egyptian population, and an association between the *PNPLA3* rs738409 GG genotype and increased serum alanine aminotransferase levels.

Our results showed that *PNPLA3* rs738409 C>G CG and GG genotype carriers in MASLD and liver fibrosis in MASLD cases had higher proportions than those in the controls (both *p* < 0.05). Logistic regression analysis for the association of *PNPLA3* rs738409 C>G with MASLD and liver fibrosis in MASLD showed statistical significance with an increased odds ratio. After adjusting for the risk factors (age, sex, BMI, WC, HDL, TG, diabetes mellitus, and hypertension), the association of *PNPLA3* rs738409 C>G with MASLD remained statistically significant with an increased odds ratio. As the severity of liver fibrosis in MASLD increased, *PNPLA3* rs738409 C>G CG and GG genotype carriers in subjects had higher proportions than CC genotype carriers (*p* = 0.003). Also, as the severity of hepatic steatosis in MASLD increases, *PNPLA3* rs738409 C>G CG and GG genotype carriers in subjects had higher proportions than CC genotype carriers (*p* = 0.001). F2-F3-F4 were positively correlated with the GG genotype (all *p* < 0.05), F2-F3 did not show statistical significance with the CG genotype, and F4 was positively correlated with the CG genotype (*p* = 0.042). S2 and S3 were positively correlated with the GG genotype (both *p* < 0.05), S2 did not show statistical significance with the CG genotype, and S3 was positively correlated with the CG genotype (*p* = 0.006). The *PNPLA3* rs738409 G allele is associated with increased systolic blood pressure (*p* = 0.029), diastolic blood pressure values (*p* = 0.015), and alanine aminotransferase levels (*p* = 0.002). Other clinical characteristics did not show statistically significant differences between *PNPLA3* rs738409 G allele carriers and non-carriers in the study population.

A meta-analysis study elucidated that polymorphisms in *TM6SF2* rs58542926 C>T may have an impact on the likelihood of developing NAFLD [22]. G et al. [20] revealed that this variant was associated with NAFLD but had no statistically significant effects on fibrosis. Another study revealed that this variant shows no significant correlation with NAFLD [23]. Anstee QM et al. and others confirmed that loss-of-function variations in *HSD17B13* rs9992651 G>A have been linked to the protective effect against NAFLD generally [18,21]. Abul-Husn NS et al. [38] reported a protective effect of *HSD17B13* rs9992651 G>A against fibrosis but essentially none against milder steatosis. A meta-analysis including 25 studies containing 6598 cases and 19,954 controls revealed a statistically significant association of NAFLD with the *GCKR* rs1260326 T>C [24].

Our study suggested that *TM6SF2* rs58542926 C>T, *HSD17B13* rs9992651 G>A, and *GCKR* rs1260326 T>C polymorphisms are not associated with the presence or severity of hepatic steatosis and liver fibrosis in MASLD in the Egyptian population (all *p* > 0.05). The association of *TM6SF2* rs58542926 C>T, *HSD17B13* rs9992651 G>A, and *GCKR* rs1260326 T>C polymorphisms with NAFLD may be modified by the change in the population [7,14,25,26]. After adjusting for the risk factors (age, sex, BMI, WC, HDL, TG, diabetes mellitus, and hypertension), F2 was negatively correlated with the *HSD17B13* rs9992651 GA genotype. The *TM6SF2* rs58542926 T allele is associated with increased fasting blood glucose levels (*p* = 0.017). Although the waist circumference value is significantly higher in MASLD patients compared with the controls, the *TM6SF2* rs58542926 T allele is associated with a decreased value of waist circumference (*p* = 0.013). Although the aspartate transaminase levels and diastolic blood pressure values are significantly higher in MASLD patients compared with the controls, the *GCKR* rs1260326 C allele is associated with decreased aspartate transaminase levels (*p* = 0.014), and diastolic blood pressure values (*p* = 0.043). Other clinical characteristics did not show statistically significant differences between allele carriers and non-carriers in the study population.

## 5. Conclusions

For the first time, our results confirm other worldwide studies for other populations for the positive association of the *PNPLA3* rs738409 C>G variant with the presence of MASLD with liver fibrosis, the severity of hepatic steatosis and liver fibrosis in MASLD, and increased alanine aminotransferase levels among Egyptian subjects. To our knowledge, this is also the first report worldwide to describe novel associations of the *PNPLA3* rs738409 G allele with increased systolic and diastolic blood pressure values. The *TM6SF2* rs58542926 T allele is associated with increased fasting blood glucose levels and a decreased value of waist circumference. The *GCKR* rs1260326 C allele is associated with decreased aspartate transaminase levels and diastolic blood pressure values. Only after adjusting for the risk factors (age, sex, BMI, WC, HDL, TG, diabetes mellitus, and hypertension) the *HSD17B13* rs9992651 GA genotype may have a protective effect from F2. These results are to be further investigated in wider, dedicated studies.

## Figures and Tables

**Table 1:** Baseline characteristics of MASLD cases and controls.

Phenotype	MASLD Cases (n = 205)	Controls (n = 187)	*p*-Value
Gender (male/female)	126/79	72/115	<0.001 ***
Diabetes (Diabetic/Prediabetic/Normal)	78/14/113	0/0/187	<0.001 ***
Hypertension (Hypertensive/Normal)	125/80	0/187	<0.001 ***
Age (years)	45.16 ± 11.25	38.37 ± 11.30	<0.001 ***
Hepatic Steatosis degree (S0/S1/S2/S3)	0/33/82/90	187/0/0/0	<0.001 ***
Liver Fibrosis degree (F0–1/F2/F3/F4)	74/90/31/10	187/0/0/0	<0.001 ***
BMI (kg/m^2^)	32.78 ± 5.82	22.95 ± 2.62	<0.001 ***
WC (cm)	112.52 ± 12.19	90.36 ± 4.10	<0.001 ***
SBP (mmHg)	119.98 ± 18.25	116.18 ± 10.08	0.011 *
DBP (mmHg)	83.86 ± 13.51	77.36 ± 5.98	<0.001 ***
ALT (U/L)	36.01 ± 27.39	15.16 ± 7.07	<0.001 ***
AST (U/L)	31.61 ± 20.25	20.48 ± 6.57	<0.001 ***
GGT (U/L)	41.89 ± 36.44	18.28 ± 7.55	<0.001 ***
FBG (mg/dL)	106.29 ± 34.65	86.51 ± 11.450	<0.001 ***
PPBS (mg/dL)	122.66 ± 37.01	104.60 ± 14.60	<0.001 ***
TC (mg/dL)	197.4 ± 45.62	169.27 ± 36.02	<0.001 ***
TG (mg/dL)	154.76 ± 79.15	91.67 ± 37.43	<0.001 ***
LDL-C (mg/dL)	128.75 ± 41.57	114.08 ± 28.73	<0.001 ***
HDL-C (mg/dL)	37.20 ± 11.19	39.51 ± 11.44	<0.001 ***

Values are presented as mean ± standard deviation. BMI, body mass index; WC, waist circumference; SBP, systolic blood pressure; DBP, diastolic blood pressure; ALT, alanine aminotransferase; AST, aspartate transaminase; GGT, gamma-glutamyl transpeptidase; FBG, fasting blood glucose; PPBS, post-prandial blood sugar; TC, total cholesterol; TG, triacylglyceride; LDL-C, low-density lipoprotein cholesterol; HDL-C, high-density lipoprotein cholesterol. *p*-value estimated by the Chi-Square test or Mann–Whitney test for categorical or non-normally distributed continuous, respectively, * <0.05; *** <0.001.

**Table 2:** Association of genotypes of *PNPLA3* rs738409 C>G, *TM6SF2* rs58542926 C>T, *HSD17B13* rs9992651 G>A, and *GCKR* rs1260326 T>C with MASLD by logistic regression analysis.

	MASLD Cases (n = 205)	Controls (Ref.) (n = 187)	Unadjusted Model		Adjusted Model for Age, Sex, BMI, WC, HDL, TG, Diabetes Mellitus, and Hypertension	
	No.	No.	*p*-Value ^1^	OR (95%CI)	*p*-Value ^2^	OR (95%CI)
*PNPLA3* rs738409 C>G						
Genotype						
CC (Ref.)	100	119		1.000		1.000
CG	83	60	0.022 *	1.646 (1.076–2.519)	0.941	1.043 (0.337–3.235)
GG	22	8	0.006 **	3.272 (1.396–7.670)	0.039 *	9.166 (1.123–74.832)
*TM6SF2* rs58542926 C>T						
Genotype						
CC (Ref.)	170	157		1.000		1.000
CT	34	30	0.8677	1.047 (0.612–1.790)	0.952	1.041 (0.282–3.844)
TT	1	0	1.000	-	1.000	-
*HSD17B13* rs9992651 G>A						
Genotype						
GG (Ref.)	150	130		1.000		1.000
GA	46	49	0.386	0.814 (0.511–1.296)	0.597	1.409 (0.395–5.025)
AA	8	3	0.223	2.311 (0.601–8.893)	0.511	2.726 (0.137–54.280)
*GCKR* rs1260326 T>C						
Genotype						
TT (Ref.)	70	62		1.000		1.000
TC	102	88	0.908	1.027 (0.658–1.602)	0.657	0.745 (0.203–2.731)
CC	31	32	0.617	0.858 (0.471–1.564)	0.556	1.796 (0.256–12.612)

Ref., reference group; OR, odds ratio; CI, confidence interval; *p*-value ^1^, and *p*-value ^2^ estimated by single logistic regression analysis and multivariable logistic regression analysis, respectively, * <0.05; ** <0.01.

**Table 3:** Association of genotypes of *PNPLA3* rs738409 C>G, *TM6SF2* rs58542926 C>T, *HSD17B13* rs9992651 G>A, and *GCKR* rs1260326 T>C in MASLD cases with liver fibrosis, MASLD cases without liver fibrosis, and controls.

	Control (Ref.) (n = 187) versus MASLD Cases with Liver Fibrosis (n = 131)		Control (Ref.) (n = 187) versus MASLD Cases without Liver Fibrosis (n = 74)	
	*p*-Value ^1^	OR (95%CI)	*p*-Value ^2^	OR (95%CI)
*PNPLA3* rs738409 C>G				
Genotype				
CC (Ref.) (n = 219)		1.000		1.000
CG (n = 143)	0.015 *	1.815 (1.121–2.940)	0.243	1.403 (0.795–2.475)
GG (n = 30)	0.001 **	4.538 (1.865–11.044)	0.560	1.451 (0.415–5.073)
*TM6SF2* rs58542926 C>T				
Genotype				
CC (Ref.) (n = 327)		1.000		1.000
CT (n = 64)	0.998	0.999 (0.544–1.836)	0.731	1.134 (0.554–2.319)
TT (n = 1)	-	-	-	-
*HSD17B13* rs9992651 G>A				
Genotype				
GG (Ref.) (n = 280)		1.000		1.000
GA (n = 95)	0.180	0.690 (0.401–1.186)	0.850	1.061 (0.574–1.961)
AA (n = 11)	0.298	2.167 (0.506–9.282)	0.252	2.600 (0.508–13.313)
*GCKR* rs1260326 T>C				
Genotype				
TT (Ref.) (n = 132)		1.000		1.000
TC (n = 190)	0.518	0.848 (0.515–1.397)	0.243	1.443 (0.780–2.668)
CC (n = 63)	0.584	0.830 (0.427–1.616)	0.855	0.923 (0.388–2.192)

Ref., reference group; OR, odds ratio; CI, confidence interval; *p*-value ^1^ and *p*-value ^2^ estimated by logistic regression analysis, * <0.05; ** <0.01.

**Table 4:** Association of genotypes of *PNPLA3* rs738409 C>G, *TM6SF2* rs58542926 C>T, *HSD17B13* rs9992651 G>A, and *GCKR* rs1260326 T>C with different severities of liver fibrosis in MASLD by logistic regression analysis.

	F0–1 (Ref.) (n = 261) VS F2 (n = 90)		F0–1 (Ref.) (n = 261) VS F3 (n = 31)		F0–1 (Ref.) (n = 261) VS F4 (n = 10)	
	*p*-Value ^1^	OR (95%CI)	*p*-Value ^2^	OR (95%CI)	*p*-Value ^3^	OR (95%CI)
*PNPLA3* rs738409 C>G						
Genotype						
CC (Ref.) (n = 219)		1.000		1.000		1.000
CG (n = 143)	0.118	1.505 (0.901–2.514)	0.297	1.241 (0.827–1.864)	0.042 *	5.393 (1.066–27.284)
GG (n = 30)	0.007 **	3.411 (1.408–8.262)	0.009 **	4.762 (1.467–15.460)	0.013 *	13.333 (1.724–103.145)
*TM6SF2* rs58542926 C>T						
Genotype						
CC (Ref.) (n = 327)		1.000		1.000		1.000
CT (n = 64)	0.978	1.009 (0.530–1.921)	0.604	0.748 (0.249–2.246)	0.774	1.262 (0.259–6.148)
TT (n = 1)	-	-	-	-	-	-
*HSD17B13* rs9992651 G>A						
Genotype						
GG (Ref.) (n = 280)		1.000		1.000		1.000
GA (n = 95)	0.097	0.596 (0.324–1.097)	0.546	0.761 (0.314–1.846)	0.713	1.304 (0.317–5.361)
AA (n = 11)	0.415	1.714 (0.470–6.258)	0.999	-	0.164	5.000 (0.518–48.295)
*GCKR* rs1260326 T>C						
Genotype						
TT (Ref.) (n = 132)		1.000		1.000		1.000
TC (n = 190)	0.393	0.797 (0.473–1.343)	0.846	0.915 (0.375–2.236)	0.106	0.253 (0.048–1.337)
CC (n = 63)	0.227	0.621 (0.287–1.345)	0.425	1.537 (0.535–4.415)	0.821	1.186 (0.270–5.202)

Ref., reference group; OR, odds ratio; CI, confidence interval; *p*-value ^1^, *p*-value ^2^, and *p*-value ^3^ estimated by logistic regression analysis, * <0.05; ** <0.01.

## Data Availability

The data presented in this study are available upon request from the corresponding author. The data are not publicly available due to privacy and ethical reasons.

## Supplementary Materials

The following supporting information can be downloaded at: https://www.mdpi.com/article/10.3390/genes15040455/s1, Figure S1: (A) Screening of Genome-wide association studies catalog for MASLD-related genetic variants identifying four genes with variants with *p*-value < 0.001. (B) Protein–protein interaction network using STRING online tool showing the interaction of the four mapped genes. The required score was set to 0.7 (high confidence). (C) Prediction of the impact of the selected genetic variants on protein function using the SIFT predictive tool. A score < 0.05 predicts the variation to be “deleterious”, whereas a score ≥ 0.05 would be “tolerated”. Figure S2: Location of *PNPLA3* gene on chromosome 22 and location of rs738409 within exon 3 with all possible alleles. Figure S3: (A) Multiple sequence alignment of PNPLA3 protein in common model organisms and domestic animals. The location of the variant (148 in human protein) is marked with a box. (B) A phylogenetic tree was conducted in MEGA X using the UPGMA method with Poisson correction. Figure S4: Comparison of the 3D structure of PNPLA3_Ile148 (A) and PNPLA3_Met148 (B) showing the main polar distances where Met148 had a larger distance compared with Ile148. Table S1: The proportions of participants with genotypes of *PNPLA3* rs738409 C>G, *TM6SF2* rs58542926 C>T, *HSD17B13* rs9992651 G>A, and *GCKR* rs1260326 T>C in MASLD cases and controls. Table S2: The proportions of participants with genotypes of *PNPLA3* rs738409 C>G, *TM6SF2* rs58542926 C>T, *HSD17B13* rs9992651 G>A, and *GCKR* rs1260326 T>C in MASLD cases with liver fibrosis, MASLD cases without liver fibrosis and controls. Table S3: Association of genotypes of *PNPLA3* rs738409 C>G, *TM6SF2* rs58542926 C>T, *HSD17B13* rs9992651 G>A, and *GCKR* rs1260326 T>C in MASLD cases with liver fibrosis, MASLD cases without liver fibrosis, and controls (After adjusting for the risk factors (age, sex, BMI, WC, HDL, TG, diabetes mellitus, and hypertension)). Table S4: The proportions of participants with genotypes of *PNPLA3* rs738409 C>G, *TM6SF2* rs58542926 C>T, *HSD17B13* rs9992651 G>A, and *GCKR* rs1260326 T>C in subjects with different severities of liver fibrosis. Table S5: Association of genotypes of *PNPLA3* rs738409 C>G, *TM6SF2* rs58542926 C>T, *HSD17B13* rs9992651 G>A, and *GCKR* rs1260326 T>C with different severity of liver fibrosis in MASLD by logistic regression analysis (After adjusting for the risk factors (age, sex, BMI, WC, HDL, TG, diabetes mellitus, and hypertension). Table S6: The proportions of participants with genotypes of *PNPLA3* rs738409 C>G, *TM6SF2* rs58542926 C>T, *HSD17B13* rs9992651 G>A, and *GCKR* rs1260326 T>C in subjects with different severities of hepatic steatosis. Table S7: Association of genotypes of *PNPLA3* rs738409 C>G, *TM6SF2* rs58542926 C>T, *HSD17B13* rs9992651 G>A, and *GCKR* rs1260326 T>C with different severity of hepatic steatosis in MASLD by logistic regression analysis. Table S8: A) Clinical characteristics of *PNPLA3* rs738409 G and *TM6SF2* rs58542926 T carriers and non-carriers in the study population. B) Clinical characteristics of *HSD17B13* rs9992651 A and *GCKR* rs1260326 C carriers and non-carriers in the study population.Table S9: A power calculation of sample size to evaluate the effect of genetic variations on MASLD.
