# Supplementary material for: Relevance of PNPLA3, TM6SF2, HSD17B13, and GCKR Variants to MASLD Severity in an Egyptian Population
Source: Genes (Basel). 2024 Apr 4;15(4):455. doi: 10.3390/genes15040455 (PMC11049340; doi:10.3390/genes15040455)
Supplement: Supplementary file 1 [file genes-15-00455-s001.zip › genes-2932796-supplementary.pdf]

**A.**

| GWAS Study | Mapped Gene | Variant | Location | p-value |
|---|---|---|---|---|
| GCST90275041 | PNPLA3 | rs738409 | 22:43928847 | $1 \times 10^{-66}$ |
| GCST90275041 | TM6SF2 | rs58542926 | 19:19268740 | $7 \times 10^{-53}$ |
| GCST90104598 | GCKR | rs1260326 | 2:27508073 | $7 \times 10^{-14}$ |
| GCST010861 | HSD17B13 | rs9992651 | 4:87311358 | $3 \times 10^{-7}$ |

**B.**

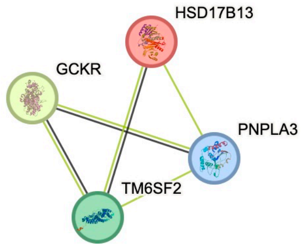


**C.**

| Protein | SNP | Variant Substitution | Substitution Score | Predicted Effect |
|---|---|---|---|---|
| PNPLA3 | rs738409 | I148M | 0.02 | Deliterious |
| TM6SF2 | rs58542926 | E167K<br>E167Ter | 0.24 | Tolerated |
| GCKR | rs1260326 | L446P<br>L446R | 0.14<br>0.31 | Tolerated<br>Tolerated |
| HSD17B13 | rs9992651 | Intron variant | - | - |

Figure S1 A) Screening of Genome-wide association studies catalog for MASLD-related genetic variants identifying 4 genes with variants with p-value < 0.001. B) Protein-protein interaction network using STRING online tool showing the interaction of the four mapped genes. The required score was set to 0.7 (high confidence). C) Prediction of the impact of the selected genetic variants on protein function using the SIFT predictive tool. A score < 0.05 predicts the variation to be “deleterious”; whereas a score ≥ 0.05 would be “tolerated”.

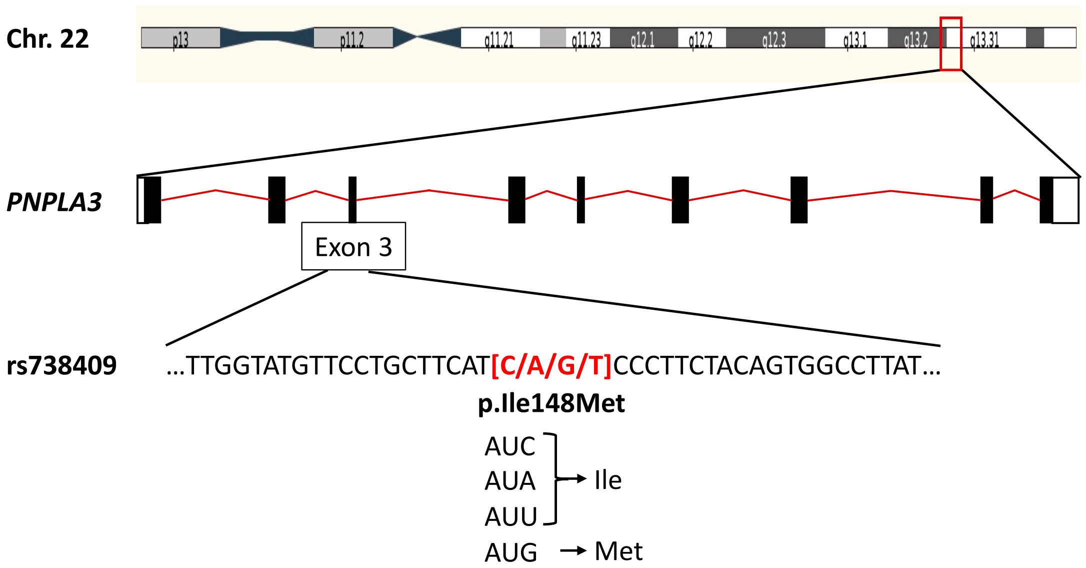


Figure S2 Location of *PNPLA3* gene on chromosome 22 and location of rs738409 within exon 3 with all possible alleles.

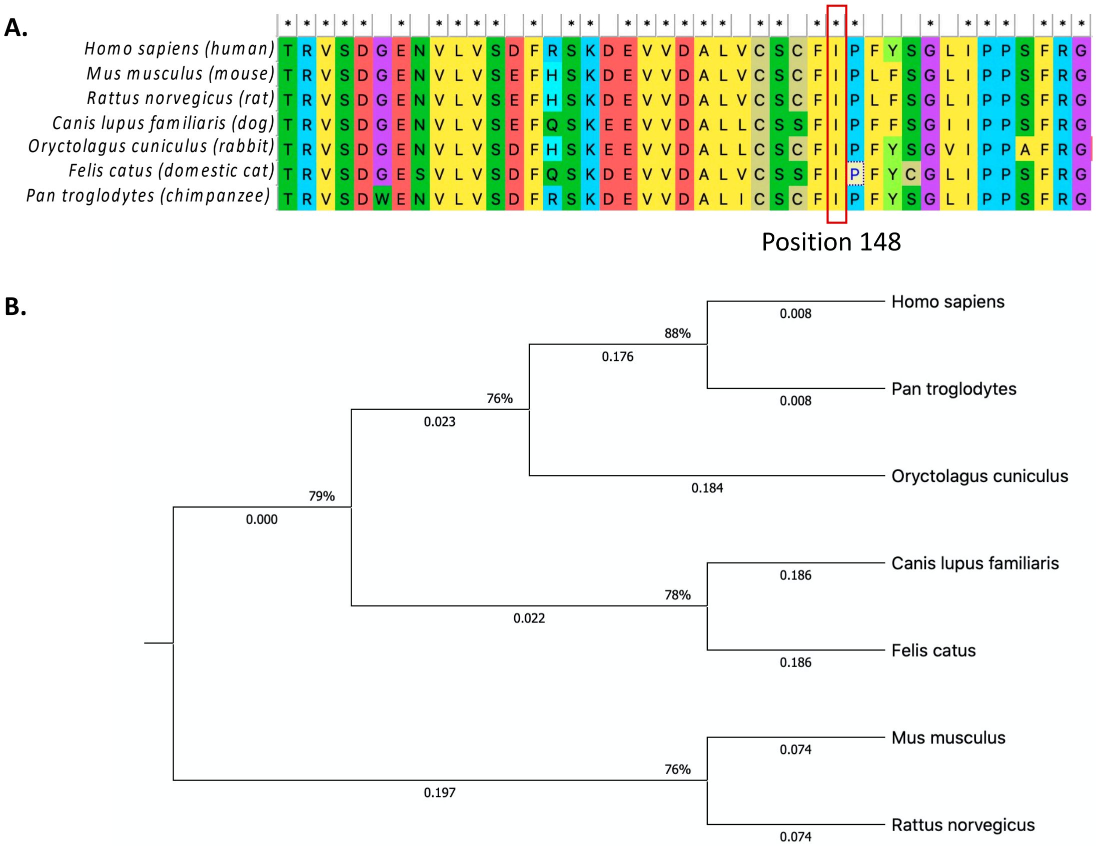


Figure S3 A) Multiple sequence alignment of PNPLA3 protein in common model organisms and domestic animals. The location of the variant (148 in human protein) is marked with a box. B) Phylogenetic tree conducted in MEGA X using UPGMA method with Poisson correction.

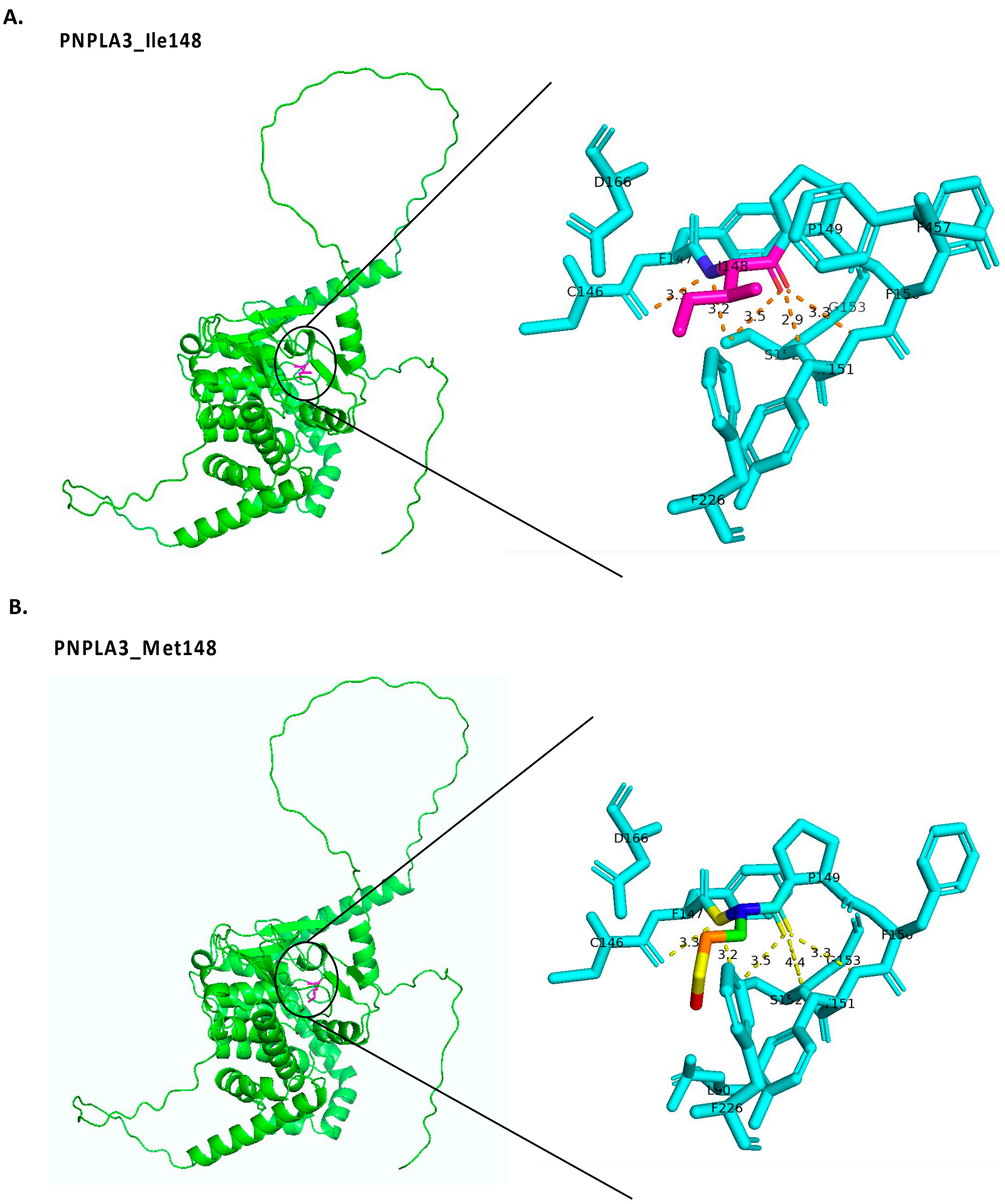


Figure S4 Comparison of the 3D structure of PNPLA3_Ile148 (A) and PNPLA3_Met148 (B) showing the main polar distances where Met148 had a larger distance compared to Ile148.

**Table S1: The proportions of participants with genotypes of *PNPLA3* rs738409 C>G, *TM6SF2* rs58542926 C>T, *HSD17B13* rs9992651 G>A, and *GCKR* rs1260326 T>C in MASLD cases and controls.**

| | MASLD cases (n = 205) | | controls (n = 187) | | P-value | FDR corrected p-value |
|---|---|---|---|---|---|---|
| | No. | % | No. | % | | |
| *PNPLA3* rs738409 C>G | | | | | | |
| Genotype | | | | | | |
| CC | 100 | 48.8 | 119 | 63.6 | 0.004** | 0.016* |
| CG | 83 | 40.5 | 60 | 32.1 | | |
| GG | 22 | 10.7 | 8 | 4.3 | | |
| $^{HW}\chi^2$(p-value) | | | 0.900 | | | |
| Allele | (n = 410) | | (n = 374) | | | |
| C | 283 | 69 | 298 | 79.7 | 0.001** | - |
| G | 127 | 31 | 76 | 20.3 | | |
| *TM6SF2* rs58542926 C>T | | | | | | |
| Genotype | | | | | | |
| CC | 170 | 82.9 | 157 | 84.0 | $^{FE}$P=0.940 | 0.940 |
| CT | 34 | 16.6 | 30 | 16.0 | | |
| TT | 1 | 0.5 | 0 | 0.0 | | |
| $^{HW}$FE (p-value) | | | 0.614 | | | |
| Allele | (n = 410) | | (n = 374) | | | |
| C | 374 | 91.2 | 344 | 92.0 | | - |
| T | 36 | 8.8 | 30 | 8.0 | 0.702 | |
| *HSD17B13* rs9992651 G>A | | | | | | |
| Genotype | | | | | | |
| GG | 150 | 73.5 | 130 | 71.4 | 0.279 | 0.558 |
| GA | 46 | 22.5 | 49 | 26.9 | | |
| AA | 8 | 3.9 | 3 | 1.6 | | |
| $^{HW}\chi^2$(p-value) | | | 0.504 | | | |
| Allele | (n = 408) | | (n = 364) | | | |
| G | 346 | 84.8 | 309 | 84.9 | 0.973 | - |
| A | 62 | 15.2 | 55 | 15.1 | | |
| *GCKR* rs1260326 T>C | | | | | | |
| Genotype | | | | | | |
| TT | 70 | 34.5 | 62 | 34.1 | 0.824 | 0.940 |
| TC | 102 | 50.2 | 88 | 48.4 | | |
| CC | 31 | 15.3 | 32 | 17.6 | | |
| $^{HW}\chi^2$(p-value) | | | 0.936 | | | |
| Allele | (n = 406) | | (n = 364) | | | |
| T | 242 | 59.6 | 212 | 58.2 | 0.701 | - |
| C | 164 | 40.4 | 152 | 41.8 | | |

p-value estimated by Chi Square test for categorical datasets, *<0.05; ** < 0.01.
$^{FE}$P Fisher's Exact corrected p-value.
$^{HW}\chi^2$: Chi square for goodness of fit for Hardy-Weinberg equilibrium.

**Table S2: The proportions of participants with genotypes of *PNPLA3* rs738409 C>G, *TM6SF2* rs58542926 C>T, *HSD17B13* rs9992651 G>A, and *GCKR* rs1260326 T>C in MASLD cases with liver fibrosis, MASLD cases without liver fibrosis and controls.**

| | MASLD cases with liver fibrosis (n = 131) | | MASLD cases without liver fibrosis (n=74) | | Controls (n = 187) | | P-value | FDR corrected p-value |
|---|---|---|---|---|---|---|---|---|
| | No. | % | No. | % | No. | % | | |
| *PNPLA3* rs738409 C>G | | | | | | | | |
| Genotype | | | | | | | | |
| CC | 59 | 45 | 41 | 55.4 | 119 | 63.6 | 0.003** | 0.012* |
| CG | 54 | 41.2 | 29 | 39.2 | 60 | 32.1 | | |
| GG | 18 | 13.7 | 4 | 5.4 | 8 | 4.3 | | |
| Allele | (n = 262) | | (n=148) | | (n=374) | | | |
| C | 172 | 65.6 | 111 | 75 | 298 | 79.7 | < 0.001 *** | - |
| G | 90 | 34.4 | 37 | 25 | 76 | 20.3 | | |
| *TM6SF2* rs58542926 C>T | | | | | | | | |
| Genotype | | | | | | | | |
| CC | 110 | 84 | 60 | 81.1 | 157 | 84 | | |
| CT | 21 | 16 | 13 | 17.6 | 30 | 16 | $^{FE}$P=0.526 | 0.526 |
| TT | 0 | 0 | 1 | 1.4 | 0 | 0 | | |
| Allele | (n = 262) | | (n=148) | | (n=374) | | | |
| C | 241 | 92.0 | 133 | 89.9 | 344 | 92 | 0.706 | - |
| T | 21 | 8.0 | 15 | 10.1 | 30 | 8 | | |
| *HSD17B13* rs9992651 G>A | | | | | | | | |
| Genotype | | | | | | | | |
| GG | 100 | 76.3 | 50 | 68..5 | 130 | 71.4 | $^{FE}$P=0.350 | 0.526 |
| GA | 26 | 19.8 | 20 | 27.4 | 49 | 26.9 | | |
| AA | 5 | 3.8 | 3 | 4.1 | 3 | 1.6 | | |
| Allele | (n = 262) | | (n=146) | | (n=364) | | | |
| G | 226 | 86.3 | 120 | 82.2 | 309 | 84.9 | 0.547 | - |
| A | 36 | 13.7 | 26 | 17.8 | 55 | 15.1 | | |
| *GCKR* rs1260326 T>C | | | | | | | | |
| Genotype | | | | | | | | |
| TT | 49 | 34.5 | 21 | 28.4 | 62 | 34.1 | 0.504 | 0.526 |
| TC | 59 | 50.2 | 43 | 58.1 | 88 | 48.4 | | |
| CC | 21 | 15.3 | 10 | 13.5 | 32 | 17.6 | | |
| Allele | (n = 258) | | (n=148) | | (n=364) | | | |
| T | 157 | 60.9 | 85 | 57.4 | 212 | 58.2 | 0.740 | - |
| C | 101 | 39.1 | 63 | 42.6 | 152 | 41.8 | | |

p-value estimated by Chi Square test for categorical datasets, ** < 0.01; *** < 0.001.
$^{FE}$P Fisher's Exact corrected p-value.

**Table S3. Association of genotypes of *PNPLA3* rs738409 C>G, *TM6SF2* rs58542926 C>T, *HSD17B13* rs9992651 G>A, and *GCKR* rs1260326 T>C in MASLD cases with liver fibrosis, MASLD cases without liver fibrosis, and controls (After adjusting for the risk factors (age, sex, BMI, WC, HDL, TG, diabetes mellitus, and hypertension)).**

| | Control (Ref.) (n = 187) versus MASLD cases with liver fibrosis (n = 131) | | Control (Ref.) (n = 187) versus MASLD cases without liver fibrosis (n = 74) | |
|---|---|---|---|---|
| | p-value [1] | OR (95%C.I) | p-value [2] | OR (95%C.I) |
| *PNPLA3* rs738409 C>G | | | | |
| Genotype | | | | |
| CC(Ref.) (n=219) | | 1.000 | | 1.000 |
| CG (n=143) | 0.783 | 1.234(0.277 -5.486) | 0.632 | 0.715 (0.181-2.828) |
| GG (n=30) | 0. 477 | 9.348 (0.020 -4454.064) | 0.043* | 9.085(1.073-76.885) |
| *TM6SF2* rs58542926 C>T | | | | |
| Genotype | | | | |
| CC(Ref.) (n=327) | | 1.000 | | 1.000 |
| CT (n=64) | 0.242 | 3.434 (0.435-27.107) | 0.594 | 0.647 (0.131 -3.201) |
| TT (n=1) | 0.995 | 0.994(0.182-5.429) | - | - |
| *HSD17B13* rs9992651 G>A | | | | |
| Genotype | | | | |
| GG(Ref.) (n=280) | | 1.000 | | 1.000 |
| GA (n=95) | 0.219 | 3.439 (0.480-24.641) | 0.940 | 0.941 (0.188-4.705) |
| AA (n=11) | 1.000 | - | 0.455 | 1.759 (0.400-7.739) |
| *GCKR* rs1260326 T>C | | | | |
| Genotype | | | | |
| TT(Ref.) (n=132) | | 1.000 | | 1.000 |
| TC (n=190) | 0.436 | 0.489(0.081-2.962) | 0.922 | 0.925 (0.191-4.474) |
| CC(n=63) | 0.414 | 0.275(0.012-6.100) | 0.353 | 2.728 (0.328-22.689) |

Ref., reference group; OR, odds ratio; C.I., confidence interval; p-value[1] and p-value[2] estimated by logistic regression analysis, *<0.05; ** < 0.01.

**Table S4: The proportions of participants with genotypes of *PNPLA3* rs738409 C>G, *TM6SF2* rs58542926 C>T, *HSD17B13* rs9992651 G>A, and *GCKR* rs1260326 T>C in subjects with different severities of liver fibrosis.**

| *PNPLA3* rs738409 C>G | | | | | |
|---|---|---|---|---|---|
| | CC n(%) | CG n(%) | GG n(%) | P value | FDR corrected p-value |
| F0-1 | 160(73) | 89(62.2) | 12(40) | $^{FE}$P=0.003** | 0.012* |
| F2 | 43(19.6) | 36(25.2) | 11(36.7) | | |
| F3 | 14(6.4) | 12(8.4) | 5(16.7) | | |
| F4 | 2(1) | 6(4.2) | 2(6.7) | | |

| *TM6SF2* rs58542926 C>T | | | | | |
|---|---|---|---|---|---|
| | CC n(%) | CT n(%) | TT n(%) | P value | FDR corrected p-value |
| F0-1 | 217(66.4) | 43(34.1) | 1(100) | $^{FE}$P=0.943 | 0.943 |
| F2 | 75(22.9) | 15(12.9) | 0(0) | | |
| F3 | 27(8.3) | 4(6.4) | 0(0) | | |
| F4 | 8(2.4) | 2(3.1) | 0(0) | | |

| *HSD17B13* rs9992651 G>A | | | | | |
|---|---|---|---|---|---|
| | GG n(%) | AG n(%) | AA n(%) | P value | FDR corrected p-value |
| F0-1 | 180(64.3) | 69(72.6) | 6(54.5) | $^{FE}$P=0.260 | 0.365 |
| F2 | 70(25) | 16(16.8) | 4(36.4) | | |
| F3 | 24(8.6) | 7(7.4) | 0(0) | | |
| F4 | 6(2.1) | 3(3.2) | 1(9.1) | | |

| *GCKR* rs1260326 T>C | | | | | |
|---|---|---|---|---|---|
| | TT n(%) | TC n(%) | CC n(%) | P value | FDR corrected p-value |
| F0-1 | 83(62.9) | 131(68.9) | 42(66.7) | $^{FE}$P=0.274 | 0.365 |
| F2 | 35(26.5) | 44(23.2) | 11(17.5) | | |
| F3 | 9(6.8) | 13(6.8) | 7(11.1) | | |
| F4 | 5(3.8) | 2(1.1) | 3(4.8) | | |

$^{FE}$P Fisher's Exact corrected p-value, *<0.05;  ** < 0.01.

**Table S5. Association of genotypes of *PNPLA3* rs738409 C>G, *TM6SF2* rs58542926 C>T, *HSD17B13* rs9992651 G>A, and *GCKR* rs1260326 T>C with different severity of liver fibrosis in MASLD by logistic regression analysis (After adjusting for the risk factors (age, sex, BMI, WC, HDL, TG, diabetes mellitus, and hypertension).**

| | F0-1 (Ref.) (n = 261) VS F2 (n = 90) | | F0-1 (Ref.) (n = 261) VS F3 (n = 31) | | F0-1 (Ref.) (n = 261) VS F4 (n = 10) | |
|---|---|---|---|---|---|---|
| | p-value[1] | OR (95%C.I) | p-value[2] | OR (95%C.I) | p-value[3] | OR (95%C.I) |
| *PNPLA3* rs738409 C>G | | | | | | |
| Genotype | | | | | | |
| CC(Ref.) (n=219) | | 1.000 | | 1.000 | | 1.000 |
| CG (n=143) | 0.585 | 1.098(0.785-1.537) | 0.606 | 1.135(0.701-1.840) | 0.299 | 1.666 (0.635 -4.372) |
| GG (n=30) | 0.008** | 5.482 (1.572-19.112) | 0.017* | 9.641 (1.493-62.233) | 0.034* | 107.296 (1.411-8157.152) |
| *TM6SF2* rs58542926 C>T | | | | | | |
| Genotype | | | | | | |
| CC(Ref.) (n=327) | | 1.000 | | 1.000 | | 1.000 |
| CT (n=64) | 0.734 | 1.152(0.510-2.604) | 0.245 | 0.455(0.121-1.717) | 0.982 | 0.976 (0.121-7.891) |
| TT (n=1) | 1.000 | - | 1.000 | - | - | - |
| *HSD17B13* rs9992651 G>A | | | | | | |
| Genotype | | | | | | |
| GG(Ref.) (n=280) | | 1.000 | | 1.000 | | 1.000 |
| GA (n=95) | 0.012* | 0.341(0.148-0.787) | 0.190 | 0.473(0.155-1.448) | 0.762 | 0.733 (0.098-5.471) |
| AA (n=11) | 0.949 | 1.030(0.414-2.561) | 0.999 | - | 0.502 | 1.933 (0.282-13.222) |
| *GCKR* rs1260326 T>C | | | | | | |
| Genotype | | | | | | |
| TT(Ref.) (n=132) | | 1.000 | | 1.000 | | 1.000 |
| TC (n=190) | 0.206 | 0.633(0.312-1.286) | 0.530 | 0.709(0.242-2.075) | 0.168 | 0.262(0.039-1.757) |
| CC(n=63) | 0.922 | 0.946(0.311-2.876) | 0.483 | 1.660(0.404-6.825) | 0.839 | 0.766 (0.059-9.968) |

Ref., reference group; OR, odds ratio; C.I., confidence interval; p-value[1], p-value[2], and p-value[3] estimated by logistic regression analysis, *<0.05; ** < 0.01.

**Table S6: The proportions of participants with genotypes of *PNPLA3* rs738409 C>G, *TM6SF2* rs58542926 C>T, *HSD17B13* rs9992651 G>A, and *GCKR* rs1260326 T>C in subjects with different severities of hepatic steatosis.**

| *PNPLA3* rs738409 C>G | | | | | |
|---|---|---|---|---|---|
| | CC n(%) | CG n(%) | GG n(%) | P value | FDR corrected p-value |
| S0-1 | 141(64.4) | 70(49) | 9(30) | 0.001** | 0.004** |
| S2 | 38(17.4) | 32(22.4) | 12(40) | | |
| S3 | 40(18.3) | 41(28.7) | 9(30) | | |
| *TM6SF2* rs58542926 C>T | | | | | |
| | CC n(%) | CT n(%) | TT n(%) | P value | FDR corrected p-value |
| S0-1 | 183(56) | 37(57.8) | 0 | | |
| S2 | 67(20.5) | 15(23.4) | 0 | $^{FE}$P=0.465 | 0.465 |
| S3 | 77(23.5) | 12(18.8) | 1(100) | | |
| *HSD17B13* rs9992651 G>A | | | | | |
| | GG n(%) | AG n(%) | AA n(%) | P value | FDR corrected p-value |
| S0-1 | 152(54.3) | 57(60) | 5(45.5) | | |
| S2 | 65(23.2) | 14(14.7) | 3(27.3) | | 0.465 |
| S3 | 63(22.5) | 24(25.3) | 3(27.3) | $^{FE}$P=0.419 | |
| *GCKR* rs1260326 T>C | | | | | |
| | TT n(%) | TC n(%) | CC n(%) | P value | FDR corrected p-value |
| S0-1 | 68(51.5) | 115(60.5) | 32(50.8) | 0.422 | 0.465 |
| S2 | 33(25) | 34(17.9) | 15(23.8) | | |
| S3 | 31(23.5) | 41(21.6) | 16(25.4) | | |

p-value estimated by Chi Square test for categorical datasets, ** < 0.01.

.

$^{FE}$P Fisher's Exact corrected p-value.

**Table S7. Association of genotypes of *PNPLA3* rs738409 C>G, *TM6SF2* rs58542926 C>T, *HSD17B13* rs9992651 G>A, and *GCKR* rs1260326 T>C with different severity of hepatic steatosis in MASLD by logistic regression analysis.**

| | S0-1 (Ref.) (n = 220) VS S2 (n = 82) | | S0-1 (Ref.) (n = 220) VS S3 (n = 90) | |
|---|---|---|---|---|
| | p-value [1] | OR (95%C.I) | p-value [2] | OR (95%C.I) |
| | | *PNPLA3* rs738409 C>G | | |
| Genotype CC(Ref.) (n=219) | | 1.000 | | 1.000 |
| CG (n=143) | 0.060 | 1.696(0.978-2.942) | 0.006** | 2.065(1.226-3.478) |
| GG (n=30) | 0.001** | 4.947(1.941-12.609) | 0.012* | 3.525(1.312-9.471) |
| | | *TM6SF2* rs58542926 C>T | | |
| Genotype CC(Ref.) (n=327) | | 1.000 | | 1.000 |
| CT (n=64) | 0.763 | 1.107(0.571-2.147) | 0.468 | 0.771(0.381-1.558) |
| TT (n=1) | - | - | - | - |
| | | *HSD17B13* rs9992651 G>A | | |
| Genotype GG(Ref.) (n=280) | | 1.000 | | 1.000 |
| GA (n=95) | 0.096 | 0.574(0.299-1.103) | 0.956 | 1.016(0.580-1.779) |
| AA (n=11) | 0.649 | 1.403(0.326-6.045) | 0.620 | 1.448(0.336-6.241) |
| | | *GCKR* rs1260326 T>C | | |
| Genotype TT(Ref.) (n=132) | | 1.000 | | 1.000 |
| TC (n=190) | 0.086 | 0.609(0.346-1.072) | 0.385 | 0.782(0.449-1.362) |
| CC(n=63) | 0.927 | 0.966(0.460-2.026) | 0.805 | 1.097(0.526-2.288) |

Ref., reference group; OR, odds ratio; C.I., confidence interval; p-value[1], and p-value[2] estimated by logistic regression analysis, *<0.05; ** < 0.01.

**Table S8. A) Clinical characteristics of *PNPLA3* rs738409 G and *TM6SF2* rs58542926 T carriers and non-carriers in the study population.**

| Phenotype | *PNPLA3* rs738409 C>G | | P-value | FDR corrected p-value | *TM6SF2* rs58542926 C>T | | P-value | FDR corrected p-value |
|---|---|---|---|---|---|---|---|---|
| | Carriers | Non-carriers | | | Carriers | Non-carriers | | |
| Diabetes (n) (Diabetic/ Prediabetic/ Normal) | (40/6/127) | (38/8/173) | 0.364 | 0.54 | (18-2-45) | (60/12/255) | 0.226 | 0.565 |
| Hypertension (n) (Hypertensive/ Normal) | (64/109) | (61/158) | 0.054 | 0.162 | (18/47) | (107/220) | 0.427 | 0.864 |
| FBG(mg/dl) | 97.23±26.901 | 96.56±28.982 | 0.935 | 0.939 | 105.68±36.151 | 95.10±25.854 | 0.017* | 0.128 |
| PPBS(mg/dl) | 115.72±33.881 | 112.73±26.457 | 0.689 | 0.939 | 115.98±25.591 | 113.66±30.772 | 0.157 | 0.565 |
| SBP(mmHg) | 119.96±17.263 | 116.75±12.854 | 0.029* | 0.145 | 118.15±14.481 | 118.17±15.152 | 0.888 | 0.982 |
| DBP(mmHg) | 81.79±10.159 | 79.95±11.717 | 0.015* | 0.113 | 80.97±12.111 | 80.72±10.885 | 0.922 | 0.982 |
| WC(cm) | 106.29±13.680 | 106.28±13.268 | 0.939 | 0.939 | 103.0±11.622 | 106.94±13.688 | 0.013* | 0.128 |
| BMI (kg/m$^2$) | 30.13±5.709 | 30.27±6.388 | 0.821 | 0.939 | 29.52±5.217 | 30.34±6.248 | 0.461 | 0.864 |
| ALT(U/L) | 30.782±27.718 | 22.333±17.377 | 0.002** | 0.030* | 27.631±24.177 | 25.750±22.652 | 0.607 | 0.911 |
| AST (U/L) | 28.961±20.710 | 24.197±11.292 | 0.052 | 0.162 | 26.508±11.899 | 26.258±17.044 | 0.200 | 0.565 |
| GGT (U/L) | 31.473±24.184 | 29.959±32.840 | 0.066 | 0.165 | 27.323±15.735 | 31.284±31.292 | 0.774 | 0.968 |
| TC(mg/dl) | 180.069±42.223 | 187.068±44.515 | 0.114 | 0.244 | 183.40±38.546 | 184.095±44.593 | 0.982 | 0.982 |
| LDL-C(mg/dl) | 119.15±36.787 | 123.805±36.619 | 0.323 | 0.538 | 123.646±32.870 | 121.374±37.472 | 0.594 | 0.911 |
| HDL-C(mg/dl) | 38.416±11.055 | 38.206±11.605 | 0.907 | 0.939 | 38.138±10.504 | 38.33±11.528 | 0.749 | 0.968 |
| TG(mg/dl) | 125.457±62.10 | 124.032±76.150 | 0.256 | 0.480 | 112.338±61.119 | 127.110±71.718 | 0.062 | 0.310 |

**B) Clinical characteristics of *HSD17B13* rs9992651 A and *GCKR* rs1260326 C carriers and non-carriers in the study population.**

| Phenotype | *HSD17B13* rs9992651 G>A | | P-value | FDR corrected p-value | *GCKR* rs1260326 T>C | | P-value | FDR corrected p-value |
|---|---|---|---|---|---|---|---|---|
| | Carriers | Non-carriers | | | Carriers | Non-carriers | | |
| Diabetes (n) (Diabetic/ Prediabetic/ Normal) | (21/5/80) | (56/9/215) | 0.780 | 0.947 | (54-6-193) | (23-8-101) | 0.141 | 0.423 |
| Hypertension (n) (Hypertensive/ Normal) | (35/71) | (90/190) | 0.870 | 0.947 | (78-175) | (45-87) | 0.515 | 0.858 |
| FBG(mg/dl) | 98.43±32.636 | 95.71±24.285 | 0.771 | 0.947 | 97.57±30.778 | 95.67±22.759 | 0.659 | 0.913 |
| PPBS(mg/dl) | 115.16±29.238 | 113.74±30.439 | 0.350 | 0.947 | 114.62±30.598 | 113.40±29.170 | 0.817 | 0.913 |
| SBP(mmHg) | 118.18±14.035 | 118.11±15.556 | 0.884 | 0.947 | 117.32±13.523 | 119.58±17.720 | 0.063 | 0.236 |
| DBP(mmHg) | 81.96±13.653 | 80.32±10.059 | 0.584 | 0.947 | 80.12±11.444 | 81.88±10.472 | 0.043* | 0.236 |
| WC(cm) | 107.01±13.264 | 106.21±13.538 | 0.749 | 0.947 | 106.38±13.820 | 106.08±12.498 | 0.902 | 0.913 |
| BMI (kg/m$^2$) | 30.51±5.956 | 30.18±6.149 | 0.524 | 0.947 | 30.21±6.308 | 30.17±5.559 | 0.913 | 0.913 |
| ALT(U/L) | 25.160±20.459 | 26.676±23.919 | 0.850 | 0.947 | 24.561±21.314 | 29.229±25.494 | 0.056 | 0.236 |
| AST (U/L) | 25.322±12.571 | 26.672±17.601 | 0.947 | 0.947 | 24.625±13.320 | 29.434±20.499 | 0.014* | 0.210 |
| GGT (U/L) | 27.858±17.036 | 31.799±32.985 | 0.879 | 0.947 | 29.735±30.705 | 32.286±27.024 | 0.269 | 0.504 |
| TC(mg/dl) | 187.679±41.059 | 182.461±44.862 | 0.246 | 0.947 | 182.237±43.759 | 187.735±43.238 | 0.243 | 0.504 |
| LDL-C(mg/dl) | 124.465±33.936 | 120.621±38.038 | 0.323 | 0.947 | 121.249±37.531 | 123.222±34.920 | 0.750 | 0.913 |
| HDL-C(mg/dl) | 38.226±11.583 | 38.190±11.255 | 0.852 | 0.947 | 38.379±11.097 | 38.198±11.499 | 0.875 | 0.913 |
| TG(mg/dl) | 133.104±85.935 | 121.921±63.63 | 0.495 | 0.947 | 119.664±60.259 | 133.303±84.558 | 0.264 | 0.504 |

Values are presented as numbers (n) or mean ± standard deviation.
BMI, body mass index; WC, waist circumference; SBP, systolic blood pressure; DBP, diastolic blood pressure; ALT, alanine aminotransferase; AST, aspartate transaminase; GGT, gamma-glutamyl transpeptidase; FBG, fasting blood glucose; PPBS, post-prandial blood sugar; TC, total cholesterol; TG, triacylglyceride; LDL-C, low-density lipoprotein cholesterol; HDL-C, high-density lipoprotein cholesterol. P-value estimated by the Chi-Square test or Mann-Whitney test for categorical or non-normally distributed continuous, respectively, *<0.05; ** < 0.01.

**Table S9. A power calculation of sample size to evaluate the effect of genetic variations on MASLD.**

| MASLD cases | controls | Test family | Total sample size | Effect size | α error probability | Achieved power |
|---|---|---|---|---|---|---|
| *PNPLA3* rs738409 C>G | | | | | | |
| n=205 | n=187 | $X^2$ | 392 | 0.3 | 0.05 | 0. 9988964 |
| *TM6SF2* rs58542926 C>T | | | | | | |
| n=205 | *n=187* | $X^2$ | 392 | 0.3 | 0.05 | 0.9988964 |
| *HSD17B13* rs9992651 G>A | | | | | | |
| n=204 | *n=182* | $X^2$ | 386 | 0.3 | 0.05 | 0.9987241 |
| *GCKR* rs1260326 T>C | | | | | | |
| n=203 | *n=182* | $X^2$ | 385 | 0.3 | 0.05 | 0.9986930 |

$X^2$ Chi Square test
